# Assessment of heavy metals and microbial contamination of *gari* from Liberia

**DOI:** 10.1002/fsn3.527

**Published:** 2017-11-20

**Authors:** Wasiu Awoyale, Robert Asiedu, William K. C. Kawalawu, Busie Maziya‐Dixon, Adebayo Abass, Michael Edet, Medinat O. Adetunji

**Affiliations:** ^1^ International Institute of Tropical Agriculture Central Agricultural Research Institute Compound Suakoko Bong County Liberia; ^2^ Department of Food Science and Technology Kwara State University Ilorin Kwara State Nigeria; ^3^ International Institute of Tropical Agriculture Ibadan Oyo State Nigeria; ^4^ Program Management Unit Ministry of Agriculture Annex University of Liberia Fendell Campus Liberia; ^5^ International Institute of Tropical Agriculture (IITA) Dar es Salaam Tanzania

**Keywords:** Cassava, contamination, gari, heavy metals, microbial, safety

## Abstract

Cassava is a staple mostly eaten in the form of *gari*, after rice in Liberia. The local method of *gari* processing often leads to product contamination, thus, a study was done to assess the heavy metals and microbial contamination of gari in eight counties of the country. A total of sixty‐one *gari* samples were collected and packaged in an airtight polyethylene bag for analyses, using standard methods. Results depict that the mean of the heavy metals in the *gari* samples is iron (Fe) 43.87 ppm, copper (Cu) 0.94 ppm, zinc (Zn) 5.49 ppm and aluminum (Al) 257.45 ppm. Yellow *gari* had the highest Fe (64.90 ppm), Cu (1.25 ppm) and Zn (7.85 ppm) content, but with the least Al content (87.15 ppm). The Fe content was lower in groundnut‐fortified *gari* (42.93 ppm), and the Cu (0.70 ppm) and Zn (3.50 ppm) content were lower in groundnut‐moringa‐fortified *gari*. The samples and counties have no significant statistical effect (*p* > .05) on the heavy metals composition of the products. No microbial growth was observed in groundnut‐fortified and groundnut‐moringa‐fortified *gari* but with coconut‐fortified *gari* having the highest total fungi count of 800 CFU/g. The major fungi identified in the *gari* samples are *Penicillium* and *Aspergillus* spps., but with their counts within the regulated level. Therefore, the gari consumed in Liberia are safe except for the high Fe and Al content, which needs to be addressed with the use of unpainted stainless steel materials as food contact surfaces.

## 1 INTRODUCTION

Cassava is the second most consumed staple food crop in Liberia after rice. It is produced by over 60% of farming households, and an important contributor to the gross domestic product. According to the Food and Agricultural Organization, 534,810 metric tons of cassava was produced in 2014 in Liberia (FAO, 2017). Fresh cassava root contains high moisture content (75%–80%), thus, undergoes rapid spoilage within 48 to 72 hr, if not immediately processed (Ashaye, Adegbulugbe, & Dawodu, 2005; Oluwole, Olatunji, & Odunfa, 2004; Oyewole & Asagbra, 2003). Processing the roots into various products increases the shelf life, and makes transportation to urban markets less expensive (Taiwo, 2006). Coulibaly, Arinloye, Faye, and Abdoulaye (2014) reported that cassava is processed into different forms in Liberia; fresh roots are processed into dumbo, wet *fufu*, starch, *depah,* and *gari* among others. The leaves are also made into soup in the fresh or dried form.

*Gari* is a dry, crispy, creamy‐white and granular product, which is produced by crushing the cassava root into a mash, fermented, dewatered, and sieved into grits. The grits are then roasted to make the gari. *Gari* is a popular cassava food in Liberia and most parts of West Africa and some countries of Central Africa. It is commonly consumed directly or soaked in cold water with sugar, coconut, roasted peanut, or boiled cowpea as compliments, or as a stiff gel made with hot water and eaten with soup or stew. The acceptance and popularity of *gari* in urban and rural areas of West and Central Africa are attributed to its ability to store well, its convenience and ready‐to‐eat form (Flach, 1990). Most of the *gari* produced in Liberia is by low‐level traditionally techniques due to a lack of modern processing equipment. The traditional cassava grater used is made of a flat iron sheet perforated with nails and fastened onto a wooden board (Coulibaly et al., 2014). The grating is done by rubbing the peeled roots against the sharp perforated surface of the iron sheet which grates the root into the mash. Bruising or injuries of the hands of the processor is common, leading to blood stains in the grated cassava. Stones and or tied woods are used to press out the excess moisture from the grated mash, however, pressers are used in some places. The roasting process is then done, using pans made from iron or earthen pots, and fire woods as a source of energy (Coulibaly et al., 2014). Additionally, partially roasted gari is completely dried in the sun without putting into consideration environmental pollution from moving vehicles.

This common traditional method of processing cassava roots in Liberia could result in poor quality products that may be contaminated by foreign matter and disease‐causing agents (Bolade, 2016). However, there is insufficient information on the safety of *gari* produced in Liberia. Therefore, this study is aimed at assessing the heavy metals and microbial contamination of *gari* produced and consumed in Liberia.

## 2 MATERIALS AND METHODS

### 2.1 Collection of gari samples in Liberia

Sixty‐one gari samples (white *gari*‐45, yellow *gari*‐1, Coconut‐fortified gari‐4, Groundnut‐fortified *gari*‐10, and Groundnut‐moringa‐fortified *gari*‐1) were collected from the processors and marketers in eight counties; Rivercess, Grand Bassa, Bomi, Margibi, Sinoe, Gbarpolu, Montserrado, and Grand Capemount, for assessment. The yellow *gari* and groundnut‐moringa‐fortified *gari* were collected from just a point in Montserrado County. Each of the *gari* samples collected is a representative of the sampling frame, thus, the unequal sampling size. Samples were packaged in hermetically sealed polyethylene bags for laboratory analyses. The processing methods for the different types of *gari* are described in Table 1.

**Table 1:** Processing methods for *gari* from fresh cassava roots in Liberia

Type of gari	Processing method
White *gari*	Peeling, washing, grating, bagging and dewatering, granulation, and roasting in earthenware pots.
Yellow *gari*	Same processing steps as above with mixing of palm oil to the granules before roasting
Coconut‐fortified *gari*	Grating and roasting of matured coconut pulp before mixing with white gari
Groundnut‐fortified *gari*	Roasting and milling of groundnuts before mixing with white gari
Groundnut‐moringa‐fortified *gari*	Drying of fresh moringa leaves, milling, and mixing with groundnut‐fortified gari

### 2.2 Analysis of heavy metal contamination

The iron, zinc, copper, and aluminum content of the samples were determined, using the method described by Jones, Benton, and Vernon (1990). The samples were ashed at 550°C. The ash was dissolved in 5 ml water and 15 ml HNO_3_/HCl (1:3) for heavy metal determination, using Atomic Absorption Spectrophotometer (Buck 205 model; Back Scientific, USA).

### 2.3 Analysis of microbial contamination

Analysis of microbial contamination by total plate count of fungi was done following the method described by Amankwah, Barimah, Acheampong, Addai, and Nnaji (2009). Fungal isolates were identified and characterized under a light microscope (Leica Galen III) based on morphological and cultural features as described by Harrigan and McCance (1976).

### 2.4 Statistical analysis

Analysis of variance (ANOVA), separation of the mean values (using Duncan's Multiple Range Test at *p* < .05), and frequency distributions were calculated, using Statistical Package for Social Scientists (SPSS) software (version 21.0).

## 3 RESULTS AND DISCUSSIONS

### 3.1 Heavy metal composition of gari products in Liberia

Dix (1981) reported that human exposure to heavy metals causes serious adverse health effects, including reduced growth and development, cancer, organ damage, and in extreme cases—death. Nevertheless, iron (Fe), copper (Cu), and zinc (Zn) are also referred to as trace metals, which are naturally present in foodstuff and confer some nutritional benefits to human, but toxic when consumed in excess (Magomya, Yebpella, Udiba, Amos, & Latayo, 2013). The means of the heavy metal composition of the *gari* samples were 43.87 ppm, 0.94 ppm, 5.49 ppm and 257.45 ppm for Fe, Cu, Zn, and Al, respectively (Table 2).

**Table 2:** Heavy metal composition of *gari* products in Liberia

Samples	N	Fe (ppm)	Cu (ppm)	Zn (ppm)	Al (ppm)
White gari	90	45.00 ± 52.86a	0.94 ± 1.00a	5.47 ± 6.49a	136.59 ± 165.13a
Coconut‐fortified *gari*	8	60.75 ± 66.58a	0.93 ± 1.00a	4.64 ± 5.38a	104.16 ± 124.83a
Yellow gari	2	64.90 ± 91.78a	1.25 ± 1.77a	7.85 ± 11.10a	87.15 ± 123.25a
Groundnut‐ fortified *gari*	20	42.93 ± 51.14a	0.93 ± 0.99a	6.04 ± 6.86a	180.52 ± 221.25a
Groundnut‐moringa‐ fortified *gari*	2	44.35 ± 62.72a	0.70 ± 0.99a	3.50 ± 4.95a	118.75 ± 167.94a
Counties					
Montserrado	52	51.89 ± 61.46a	0.92 ± 0.98a	4.92 ± 5.32ab	105.01 ± 179.45b
Bomi	6	25.62 ± 29.31a	0.90 ± 1.01a	4.07 ± 4.67b	1112.85 ± 128.02a
Gbarpolu	6	25.80 ± 29.40a	1.00 ± ± 1.16a	10.88 ± 14.85a	138.68 ± 154.91b
Grand Bassa	20	25.36 ± 29.74a	0.91 ± 0.96a	4.76 ± 5.65ab	145.60 ± 151.80b
Rivercess	4	50.20 ± 58.68a	1.00 ± 1.15a	5.80 ± 7.07ab	141.92 ± 165.13b
Margibi	20	55.65 ± 59.92a	0.88 ± 0.93a	6.10 ± 6.65ab	110.00 ± 143.34b
Grand Cape mount	12	47.29 ± 51.79a	0.87 ± 0.93a	5.49 ± 6.11ab	177.98 ± 274.55b
Sinoe	20	37.36 ± 42.29a	1.10 ± ± 1.17a	5.85 ± 6.21ab	127.53 ± 131.09b
Mean		43.87	0.94	5.49	257.45
P Samples		NS	NS	NS	NS
P Counties		NS	NS	NS	NS
P Samples × Counties		NS	NS	NS	NS

P, phosphorus; Fe, iron; Cu, copper; Zn, zinc; Al, aluminum; NS, not significant.

Means with different letters along the same column are significantly different (*p *< .05).

Although there were no statistically significant differences (*p* > 0.05) in the heavy metal composition of the products (Table 2), yellow *gari* had the highest Fe (64.90 ppm), Cu (1.25 ppm), and Zn (7.85 ppm) content, but the least Al content (87.15 ppm). The Fe content was lower in groundnut‐fortified *gari* (42.93 ppm), and the Cu (0.70 ppm) and Zn (3.50 ppm) content were lower in groundnut‐moringa‐fortified *gari*. Additionally, groundnut‐fortified *gari* had the highest Al content (180.52 ppm).

Considering the Counties, there were no statistically significant differences (*p* > .05) in the heavy metal compositions of the products apart from Zn (Table 2). Products from Margibi had the highest Fe (55.65 ppm) content, Bomi had the highest Al (1,112.85 ppm) and the lowest Zn (4.07 ppm) content and Gbarpolu and Sinoe had the highest Zn (10.88 ppm) and Cu (1.10 ppm) content, respectively. The least Fe (25.36 ppm) and Cu (0.87 ppm) content were found in Grand Bassa and Grand Capemount counties, respectively. Montserrado County has the lowest Al (105.01 ppm).

Metals such as Fe, Cu, and Zn have been observed to be essential components of many alloys, wires, and vehicle tyres and, which are usually released into the roadside environment because of mechanical abrasion and normal wear and tear (Harrison, Laxen, & Wilson, 1981). This implied that yellow *gari* with the highest values of these metals might have been roasted closer to a roadside or a market where heavy vehicular movement exist. However, the values for Zn and Cu in the yellow *gari* were below the recommended maximum limit of 10 mg/100 g and 7.3 mg/100 g respectively, stipulated by the Food and Agricultural Organization (FAO), and World Health Organization (WHO), while the Fe content of the sample was higher than the FAO/WHO standard of 42.5 mg/100 g (FAO/WHO, 2001). Thus, it will be important for the processors in Margibi counties with the highest Fe contained *gari*, to replace the local graters and or roaster made with galvanized or mild steel with stainless steel machines to reduce Fe contamination. Additionally, *gari* roasting should be completed on the roasting pan and not further dried under the sun closer to heavy vehicular movement (Bolade, 2016). Furthermore, the higher Al content in relation to the lower Fe and Zn content in the gari samples may be attributed to contamination caused by the water used during processing or migration from the paints used on the food contact surfaces/processing machines (Nanda, Biswal, Acharya, Rao, & Pujari, 2014; Sato et al., 2014; Stahl et al., 2017). The Al content of the gari samples is very high compared to the stipulated provisional tolerable weekly intake of 7 mg/kg body weight reported by the Joint FAO/WHO Expert Committee on Food Additives (JECFA) and the Scientific Committee for Food (SCF) (JECFA, 1989). Thus, food contact surfaces/machines should not be painted with metallic polish to reduce Al contamination, as accumulation of Al has been reported to be potentially cyto‐ and neurotoxic to humans (Austrian Department of Health, 2014).

### 3.2 Microbial identification in gari products

The total fungi count (TFC) of the *gari* samples revealed that no microbial growth was observed in groundnut‐fortified and groundnut‐moringa‐fortified *gari,* but with coconut‐fortified *gari* having the highest TFC of 800 CFU/g (Table 3). Additionally, *gari* from Bomi and Grand Cape mount counties have no fungi growth, but with *gari* from Rivercess county having the highest TFC (1500 CFU/g) (Table 3). The TFC of the *gari* samples in the present study is lower compared to the result of Obadina, Oyewole, and Odusami (2009) and Olopade, Oranusi, Ajala, and Olorunsola (2014). Obadina et al. (2009) reported 1.79 × 10^4^ CFU/g for the TFC of *gari* collected from markets in Abeokuta Ogun state, while Olopade et al. (2014) reported the TFC of *gari* from different markets in Ota, Ogun state Nigeria to be, from no growth to 3,000 CFU/g. The International Commission on Microbiological Specifications for Foods (ICMSF) stated that ready to eat foods with counts of ≤10^3^ CFU/g are acceptable, counts of 10^4^–10^5^ CFU/g are tolerable while counts ≥10^6^ CFU/g are unacceptable (ICMSF, 1996). Thus, since the average TFC value of the *gari* samples is <10^3^ CFU/g, they may be safe for consumption.

**Table 3:** Microbial counts and identification in gari products

Samples	Total fungi count (CFU/g)	Organism identified
White *gari*	525.00 ± 823.46a	*Penicillium* sp.*, Macrophomina* sp., *Aspergillus* sp.
Coconut‐fortified *gari*	800.00 ± 663.32a	*Penicillium* sp.*, Aspergillus* sp.
Yellow *gari*	342.00 ± 310.55a	*Aspergillus flavus, Penicillium* sp., *Rhizopus* sp.
Groundnut‐fortified *gari*	NG	NI
Groundnut‐moringa‐fortified *gari*	NG	NI
Counties		
Montserrado	479.31 ± 794.80ab	*Penicillium* sp.*, Macrophomina* sp.
Bomi	NG	NI
Gbarpolu	166.67 ± 288.68b	*Aspergillus flavus, Penicillium* sp.
Grand Bassa	980.00 ± 1,137.05ab	*Rhizopus* sp.
Rivercess	1,500.00 ± 707.11ab	*Penicillium* sp.
Margibi	866.67 ± 837.02ab	*Aspergillus niger, Aspergillus flavus, Penicillium* sp.
Grand Cape Mount	NG	NI
Sinoe	208.33 ± 2,429.30a	*Aspergillus niger, Aspergillus flavus, Penicillium* sp.
Mean	812.99	
P Samples	NS	
P Counties	NS	
P Samples × Counties	NS	

NG, no fungi growth; NI, no fungi identified; NS, not significant.

Means with different letters along the same column are significantly different (*p *< .05).

Fungi such as *Penicillium* and *Aspergillus* spps. were found in white, yellow, and coconut‐fortified *gari,* with *Macrophomina* sp. detected in only white *gari* (Table 3). *Rhizopus* spp. was found in *gari* from Grand Bassa county only. However, there is need to reduce the presence of *Aspergillus* and *Penicillium* spps. in the *gari* samples by following strictly good manufacturing and hygienic practices as these microorganisms are known to produce harmful mycotoxins (Kabak, Dobson, & Var, 2006; Oranusi, Wesley, & Oguoma, 2013). The products have no statistically significant effect (*p* > .05) on the TFC.

## 4 CONCLUSION

The *gari* produced in Liberia may be safe for consumption since the zinc and copper content were below the recommended maximum limit stipulated by the FAO/WHO. However, there is a need for caution in the use of mild or galvanized steel instead of stainless steel materials as food contact surfaces, as well as painting of processing machine surfaces with metallic polish because of the high iron and aluminum content in the *gari*. Although the average total fungi count value of the *gari* samples is less than the International Commission on Microbiological Specifications for Foods levels, there is need to reduce the presence of *Aspergillus* and *Penicillium* spps. in the *gari* samples by following strictly good manufacturing/hygienic practices, as these microorganisms are known to produce harmful mycotoxins under favorable conditions.

## CONFLICT OF INTEREST

No conflict of interest.
